# Antiplatelet and Anticoagulation Therapy in Athletes: A Cautious Compromise… If Possible!

**DOI:** 10.3390/jcdd12040151

**Published:** 2025-04-10

**Authors:** Flavio D’Ascenzi, Guglielmo Leonardo Manfredi, Vincenzo Minasi, Gian Luca Ragazzoni, Luna Cavigli, Alessandro Zorzi, Giulia Elena Mandoli, Maria Concetta Pastore, Marta Focardi, Matteo Cameli, Massimo Fineschi, Serafina Valente

**Affiliations:** 1 Sports Cardiology and Rehab Unit, Department of Medical Biotechnologies, University of Siena, 53100 Siena, Italy; 2 Division of Cardiology, Policlinico Tor Vergata, University of Rome Tor Vergata, 00133 Rome, Italy; 3 Department of Cardiac, Thoracic and Vascular Sciences and Public Health, University of Padova, 35122 Padova, Italy; 4 Department of Medical Biotechnologies, Division of Cardiology, University of Siena, 53100 Siena, Italy; 5 Division of Interventional Cardiology, University Hospital Santa Maria alle Scotte, 53100 Siena, Italy

**Keywords:** sports cardiology, antithrombotic therapy, athletes, antiplatelet therapy, anticoagulant therapy

## Abstract

Antiplatelet and anticoagulation therapy are commonly used in the general population and sometimes in athletes experiencing cardiovascular disorders. In these cases, the treatment has to be tailored according to the individual bleeding and thrombotic risk profile, also considering the intrinsic risk of sports activities when advising athletes for eligibility for competitive sports. In athletes, it is necessary to pre-assess the individual bleeding risk, considering not only the personal bleeding risk (usually low in athletes) but also the type of sport the athlete would like to practice, with careful consideration in sports where traumatic collisions are highly likely. Additionally, non-steroidal anti-inflammatory drugs are commonly used among athletes, and antiplatelet therapy may further increase the bleeding risk. Therefore, in selected competitive athletes, the default approach for antithrombotic therapy could be personalized. This review discusses the clinical management challenges of competitive athletes under antithrombotic or antiplatelet therapy, focusing on the intrinsic risks of sports practice and the indications for sports eligibility and disqualification.

## 1. Introduction

Antiplatelet and anticoagulation drugs are commonly used in cardiac patients, particularly those at high risk of thrombus formation, with clear benefits in cardiovascular (CV) outcomes [1,2]. Since some patients may practice competitive and non-competitive sports, tailoring their usage to their individual bleeding and thrombotic risk profile is essential. Athletes may have a higher bleeding risk due to several sport-related factors. High-impact or contact sports inherently carry a greater risk of trauma, which can lead to both external and internal bleeding, especially in those on antithrombotic therapy. Additionally, repetitive microtraumas, common in endurance sports, may predispose athletes to soft tissue, musculoskeletal bleeding, or even hematological disorders.

Furthermore, in the athletic population, some peculiarities should be considered for a tailored management of single (SAPT) and double antiplatelet therapy (DAPT) or direct oral anticoagulant (DOAC) therapy, such as the appropriate or inappropriate use of non-steroidal anti-inflammatory drugs (NSAIDs), which are generally taken for their anti-inflammatory/analgesic properties. However, their consumption is associated with a well-known antiplatelet action and an inherent increased risk of bleeding, particularly in patients already on SAPT or even DAPT or DOAC therapy [3].

Some authors have pointed out that competitive athletes could benefit from modifying the typical dosing of anticoagulants recommended for the general population to lower the risks of adverse effects related to major bleeding while maintaining a similar efficacy [4,5]. However, this approach should be balanced with the need to offer the best therapy possible, irrespective of athletic status, when an indication of antiplatelet or anticoagulation treatment exists.

This review discusses the clinical management challenges of competitive athletes on anti-thrombotic or antiplatelet therapy, focusing on the intrinsic risks of sports practice and the indications for sports eligibility and disqualification.

### 1.1. Chronic Coronary Syndromes

Coronary artery disease (CAD)—now renamed chronic coronary syndrome (CCS)—is currently one of the major causes of premature death in the general population. Young people and adults, including those who are engaged in regular physical activity, are not immune from dying suddenly because of CCS, particularly in the case of sedentary habits [6]. Therefore, this topic is particularly interesting for sports cardiology because the population of non-professional master athletes (athletes > 35 years old)—a subgroup of athletes at risk of major adverse cardiovascular ischemic events—is growing, especially in mass endurance events [7,8]. Regarding pharmacological management of CCS, current ESC guidelines (2024) [9] recommend SAPT for primary prevention with acetylsalicylic acid (ASA) or Clopidogrel, whereas, in the case of high ischemic risk, revascularization followed by DAPT for 6 months (ASA plus Clopidogrel). In a selected portion of patients with low ischemic risk and high risk of bleeding, it is possible to shorten DAPT to 1–3 months followed by SAPT (possibly a P2Y12 antagonist), according to both the American Heart Association (AHA) and the ESC guidelines [9,10,11].

**Figure 1:**
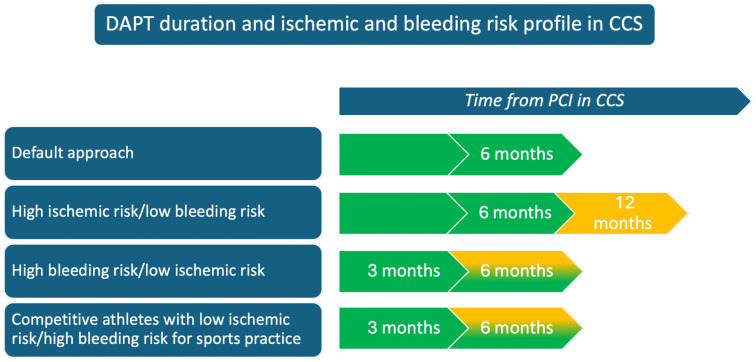
Double antiplatelet therapy (DAPT) duration according to the risk profile in chronic coronary syndromes (CCS).

During DAPT, sports with a high risk of trauma must be avoided due to the increased hemorrhagic risk. Guidelines and protocols recommend suspending activities with a likelihood of significant impacts, given the elevated risk of bleeding complications [12]. Therefore, DAPT duration could be adapted in selected cases, and, in cases of low ischemic risk, DAPT could be shortened to allow for a safe return to sports practice. Existing scores may help clinicians evaluate the athlete’s thrombotic risk after PCI. However, the possibility of changing the default approach for antithrombotic therapy in athletes should be balanced with the benefits associated with standard treatment and should be a case-by-case decision, with shared informed consent with the patient (see Figure 1).

The evidence remains controversial for SAPT, as there is limited direct scientific data or formal guidelines specifically advising against its use during sports practice [12,13]. Furthermore, since SAPT can involve either ASA or Clopidogrel, specific recommendations may vary based on the medication used. More stringent counseling is advised for ASA, given that its bleeding complications are both more severe and more frequent than those associated with Clopidogrel [14]. Therefore, a shared decision-making (SDM) approach would be reasonable in these cases since it is especially suited for these gray zones where clear answers are not provided. However, in some countries (see Italy), protocols for competitive sports eligibility exist (COCIS), providing guidance for physicians evaluating competitive athletes but also constituting a legal framework that restrains the possibility of an SDM approach, particularly if the sports physician is entirely responsible for the eligibility [12].

### 1.2. Acute Coronary Syndromes

Acute coronary syndromes (ACSs) are a spectrum of urgent CV conditions that range from unstable angina to ST elevation-ACS. These conditions can affect even the most athletic individuals, partly because physical training can, at high intensity, trigger an acute CV event [15,16] and partly because the practice of regular physical activity accounts for only a small part of the general health of an individual [17].

In terms of antiplatelet therapy, after the acute event, the 2023 ESC guidelines [18] recommend DAPT for 12 months (aspirin + a potent P2Y12 receptor inhibitor such as prasugrel or ticagrelor) as the default follow-up strategy. Randomized controlled trials have demonstrated the possibility to shorten DAPT in selected populations with a high bleeding risk [19,20,21]. In patients with low ischemic risk, the possibility of shortening DAPT duration from 12 to 3–6 months has been examined. While initially, a greater number of ischemic events occurred with a 3-month DAPT, with the advent of new-generation DES, the possibility of shortening DAPT has been proved to be feasible, reducing the bleeding risk without augmenting the thrombotic risk according to the TICO randomized controlled trial [22].

However, currently, the European and US guidelines [18,23] do not recommend this strategy as the default approach in the entire ACS population, but it can be considered whereby specific criteria justify the approach. The existing risk score may be used to assess the thrombotic risk better. It should be considered that the TICO trial [22] was conducted with ticagrelor, a strong antiplatelet drug that, compared to a potential SAPT with Clopidogrel or ASA, may exhibit a more relevant bleeding risk.

Nonetheless, the duration of DAPT should be lengthened in individuals at very high ischemic risk and without a high bleeding risk [18,24].

While participation in most competitive sports should be suspended during DAPT, after an ACS, it is highly recommended that a structured exercise program in the cardiac rehabilitation setting be prescribed, aiming to improve the CV risk profile and outcomes [18,25]. Regarding sports practice, return to play (RTP) should not be immediate after the event since there is an increased risk of ventricular arrythmias in the following months after an ACC. For this reason, the rehabilitation program may last from 6 to 12 months, depending on the clinical presentation and characteristics [26,27]. During the initial period, rehabilitation recommendations should be individualized and specific information on how to progress the volume and intensity of physical activities should be given, with an exercise intensity that should be adapted individually [28].

The different national and international guidelines for sports eligibility/disqualification vary widely, mainly because recommendations rely on experts’ opinions rather than solid evidence. Some guidelines recommend carefully evaluating athletes after an acute event, including echocardiography and cardiopulmonary exercise testing before the RTP [28,29]. Some others disqualify from sports competitions all athletes experiencing ACS and suggest a 12-month DAPT with an indication to receive default antithrombotic treatment [12].

**Figure 2:**
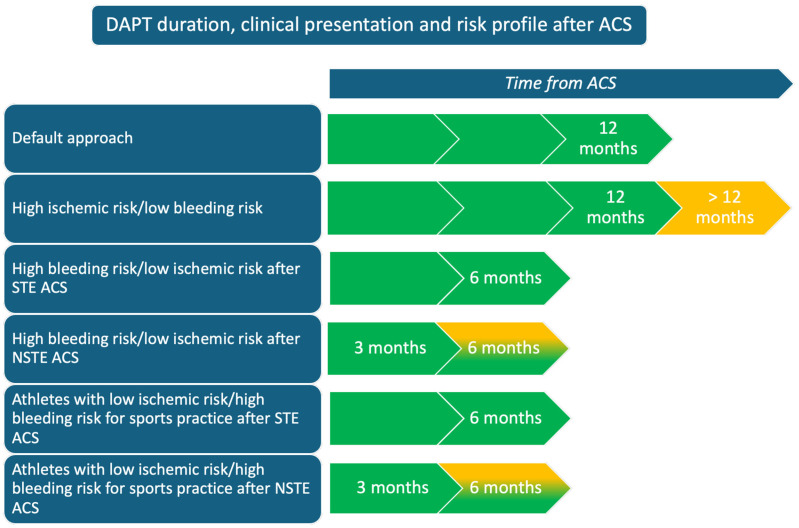
Double antiplatelet therapy (DAPT) duration according to clinical presentation and risk profile after an acute coronary syndrome (ACS). Figure legend: STE ACS, ST-elevation acute coronary syndrome; NSTE ACS, non-ST elevation acute coronary syndrome.

Considering this, after ACS athletes should receive the conventional antithrombotic regimen recommended for the general population. However, in light of the recent studies, since athletes usually present a plausible low ischemic and bleeding risk (due to the patient’s characteristics), it may be reasonable in selected cases, after a careful, comprehensive analysis based on the clinical characteristics, to consider shortening the duration of DAPT to facilitate a faster and safer RTP (see Figure 2 and Table 1). At the same time, we should consider that, after an ACS, many athletes are no longer eligible to practice competitive sports (except for some sports with low CV demand, in selected cases), and sports cardiologists and sports physicians should focus on a tailored exercise prescription in these individuals [30].

### 1.3. Antiplatelet Therapy After Atrial Septal Defect and Patent Foramen Ovale Closure

Atrial septal defects (ASDs) and patent foramen ovale (PFO) are common congenital disorders, with specific clinical indications for closure only in selected cases. In individuals with ASDs, according to the ESC guidelines (2020) [31], the indication of closure is typically based on right ventricle impairment, suspicion of paradoxical embolism or when pulmonary vascular resistance is high with a significant left-to-right shunt (Qp/Qs > 1.5), either with percutaneous or with surgical intervention.

Regarding PFO, the defect is usually small and not associated with relevant clinical signs or symptoms. Closure is usually recommended in case of cryptogenic stroke in individuals <60 years old [32]. In the absence of a hemodynamic impact of ASD and in the case of a small PFO not associated with symptoms or previous cerebrovascular events, the athletes are eligible for all competitive sports except for scuba diving [12,27,33]. However, a yearly follow-up (medical examination comprehensive of echocardiography and Holter monitoring) is recommended for athletes with ASD, particularly in case of changes in training practice, including the type of sports, training volume, and intensity that may affect the hemodynamics of the heart and change the impact of the defect [34].

After ASD and PFO closure, irrespective of surgical or percutaneous correction, DAPT duration ranges from 1 month to 6 months after the procedure, followed by a maximum of 5 years of SAPT, having the same consequences and clinical reasoning for eligibility and bleeding risk aforementioned [35].

**Figure 3:**
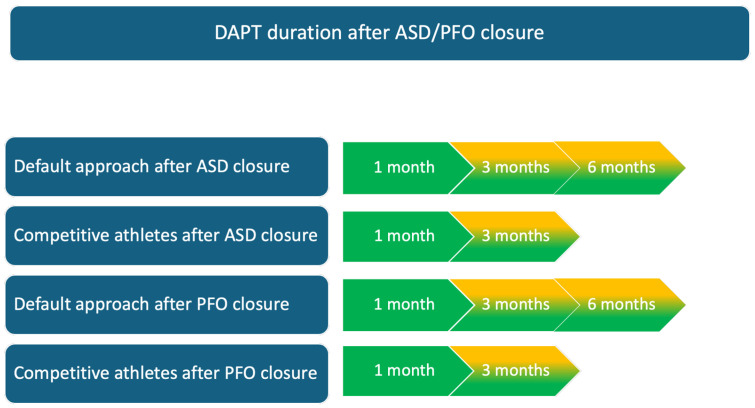
Double antiplatelet therapy (DAPT) duration after patent foramen ovale (PFO) or atrial septal defect (ASD) closure.

The follow-up differs between ASD and PFO closure. Indeed, after ASD correction, it is important to demonstrate a hemodynamic improvement of the right ventricle, the absence of arrhythmias and normal pulmonary artery pressure. In athletes who have undergone a successful procedure, returning to competition should be assessed on a case-by-case basis, typically within 3 to 6 months (see Figure 3) [12,36].

After PFO closure, residual shunts should be investigated. In their absence, the athlete is eligible for all competitive sports. At the same time, scuba diving is not allowed in case of residual shunts due to a risk of paradoxical embolism [12,37]. Although free diving is not explicitly prohibited in any guidelines, it is important to note that a residual shunt may elevate the risk of decompression sickness in these patients, even during free diving [38]. Regarding antiplatelet therapy, SAPT should be continued in case of a residual shunt after PFO closure, while it can be stopped after a successful procedure.

After ASD correction, the choice to maintain or not SAPT is still controversial and remains without a definitive, mandatory indication, opening the way to a collaborative approach (SDM) between patients and healthcare providers, especially for those practicing high-risk collision sports.

Additionally, in athletes—particularly those practicing contact sports—the dislocation of the device should also be considered, although infrequent, as it can lead to the occlusion of the peripheral vascular system [39,40]. Therefore, when the athlete is eligible for competitive sports, a strict follow-up is mandatory if the athlete is engaged in contact sports. In these cases, even if DAPT and SAPT should follow the main clinical indications recommended for the general population, they can be shortened, when possible, to allow a safe sports practice (see Table 1).

### 1.4. Anticoagulation Therapy and Sports Eligibility in Athletes with Atrial Fibrillation

Although atrial fibrillation (AF) is relatively common in the population of elderly patients with structural and valvular heart disease [41], it can also be observed in competitive athletes, particularly male master athletes practicing endurance and ultra-endurance sports, even when they do not have a structurally abnormal heart (lone AF) [42,43]. Therefore, while moderate-intensity, regular physical activity is a cornerstone in preventing AF by modifying many of its predisposing factors and patients at risk of AF should be motivated to exercise, high-intensity endurance sports significantly impact the occurrence of AF [44].

Usually, AF is present in competitive athletes as a solitary condition—and in its sporadic form—with no increased risk of stroke. Antithrombotic therapy is usually not indicated in this specific population, even if it should be weighed on the risk/benefit ratio and calculated using validated clinical assessment tools (e.g., CHA2DS2-VASc and HAS-BLED), as recommended for the general population, with inherent consequences for sports and disqualification. Nevertheless, when using these tools, it should be noted that they have not been validated on athletes.

In case anticoagulation therapy is indicated, according to the latest ESC guidelines (2024), direct oral anticoagulants (DOACs) should be preferred over vitamin K antagonists, except for prosthetic heart valves [45]. Routinely, the administration is daily, even though a recent pilot study evaluated the possibility of an intermitting-dosing strategy in patients with paroxysmal AF, with promising results [46].

In athletes, when AF is associated with a high ventricular response and relevant symptoms (pre-syncope or syncope) and when an indication for DOACs exists, the intrinsic traumatic risk should be carefully considered for each sports discipline; for instance, if the athlete practices air sports, such as skydiving or hang gliding, the eligibility is usually not allowed because of the potentially life-threatening consequences of the symptoms associated with AF or the potential trauma in an athlete under anticoagulation therapy. Therefore, sports with direct bodily contact or prone to trauma are not recommended for individuals with AF who are anticoagulated (see Table 1) [28]. Notably, thanks to the peculiar properties of DOACs and especially their reduced half-life, in the future, the exploitation of a “therapeutic window”, a time interval where DOAC’s blood concentration is low enough to allow for safe sports practice in contact sports, will be considered, therefore opening new possibilities for competitive athletes [47].

ESC and new ACC guidelines do not restrict athletes with AF. Therefore, they may still be eligible for competitive sports, provided that a satisfactory rate control has been achieved. However, more restrictive guidelines such as the Italian COCIS guidelines, only allow athletes with paroxysmal AF to take part in highly intensive competitive sports, while, in the case of permanent AF, only sports with low and moderate cardiovascular demand should usually be recommended [12]. However, it should be noted that there are no in-depth studies or case studies regarding the increased frequency and severity of bleeding events during sports practice in athletes on anticoagulation therapy. Nevertheless, these events remain plausible, as contact sports, in particular, may expose even non-anticoagulated athletes to significant bleeding risks.

AF ablation should be included among the therapeutic options for athletes, as it demonstrates a high success rate in this population [48,49,50]. According to current European guidelines, AF ablation is recommended for exercising individuals with recurrent, symptomatic AF and/or those who prefer to avoid drug therapy. However, it is important to note that anticoagulation therapy must be continued for at least two months post-ablation, even with a CHA2DS2-VASc score equal to 0, which may delay the timeline for a safe return to play [28].

### 1.5. Anticoagulation Therapy with Venous Thromboembolism

Deep venous thrombosis (DVT) is not a specific sports-related injury; on the contrary, it can arguably be reduced in incidence with sports practice. However, this review briefly summarizes the therapeutic approach since it is usually treated with the same antithrombotic drugs we mentioned before, facing the same risks and challenges for competitive athletes.

European guidelines (2019) [51], suggest that, in non-cancer patients, DOACs are generally preferred as first-line therapy. Usually, treatment is divided into three phases, each corresponding to a specific time range starting from the diagnosis: the initiation phase (first 5–21 days), the long-term phase (first 3 months), and the extended phase (>3 months). The dosage and posology used depend on the specific type of drugs chosen, but it is a general rule that the dosages are downscaled as time passes. As usual, drugs are used, weighing the risk–benefit ratio, ensuring that the potential benefits outweigh the associated risks. Therefore, it is possible and sometimes advised to stop (after 3 months) these drugs altogether, especially for DVTs caused by transient risk factors [52,53]. The inherent implications for a safe sports practice should be assessed accordingly, with the same limitations exposed before for athletes with AF who are anticoagulated.

Intermitting dosing strategies have been proposed to accelerate the return to play of some athletes in the extended phase (i.e., after 3 months), based on the premise that venous thromboembolism (VTE) daily risk is very low [4,26]. Although unsuitable for every athlete, this personalized posology takes advantage of the pharmacokinetics characteristics of DOACs, which have a fast on/fast off action [4]. Even if this approach appears biologically plausible and safe, it is still necessary to collect sufficient data, especially regarding the DOAC’s equivalence, the frequency of daily administration (e.g., once or twice a day), and the implementation of an antidote before suggesting this modality as the first-line strategy for competitive athletes (see Table 1) [5].

### 1.6. Hemorrhagic Risk in the Athletic Population: From the Individual to the Sport-Specific Risk

It is well recognized that sports practice can be a risk factor for modest and severe bleeding, especially in people taking antithrombotic drugs [54], particularly in the context of high-contact sports [55]. The risks posed by some sports activities can be even catastrophic, leading to life-threatening events [56,57,58].

Therefore, before participating in competitions or training, it is necessary to pre-assess the individual bleeding risk of the player, considering not only the personal bleeding risk (usually low in young athletes) but the type of sport the athlete would like to practice.

Indeed, the athlete’s bleeding risk is mainly composed of two parts: the personal risk and the sport-specific risk. The former is essentially due to genetic factors, such as blood clotting disorders (von Willebrand Disease, Hemophilia A or B, Glanzman Thrombasthenia, factor V Leiden), comorbidities (rare in this population), and drug use; the latter is related to the sport practiced, and it is determined by several factors, like the type and frequency of collisions, the utilization of different protections and the hardness of the soil or the ball. The sum of the two provides a reliable source for making a sound assessment of the athlete’s bleeding risk, guiding its sports eligibility.

A careful analysis of the hemorrhagic risk of an athlete should also consider the possibility of concussions. They are defined as transient neurometabolic crises of the brain, triggered by a direct or indirect head trauma, that can be even life-threatening if cerebral bleeding occurs [59]. According to a recent systematic review reporting the concussion rate in team sports, men’s rugby was associated with the greatest number of events during the match and training practice, followed by men’s tackle football and women’s ice hockey [60]. Therefore, particularly in these sports, it is important to determine a reliable personal risk of bleeding since the risks of an incorrect stratification can lead to severe clinical events (see Table 1).

Furthermore, antithrombotic drugs increase the risk of muscle hematoma, especially when they are taken for long periods and under high dosages [61,62]. Muscle hematoma is a frequent lesion in collision sports—after contusions or lacerations—and athletes can have different levels of bleeding risk. Therefore, based on the factors mentioned above, it is important to also consider these events, taking into account that they can also be misdiagnosed [63] and that may have a relevant impact in the career of professional athletes.

### 1.7. Use and Abuse of NSAIDs and Steroids in Athletes

NSAIDs are commonly used among athletes, primarily since they provide a subjective relief from musculoskeletal pain and may accelerate return to function after injury, although no ergogenic effect has been demonstrated [64]. NSAIDs are typically used in contact sports, although their usage is similarly high in other non-directly traumatic sports [65]. Far from harmless, these drugs have different side effects, ranging from kidney impairment and gastric ulcers to hypertension and even ACS [66,67]. For the scope of this review, it is nonetheless important to focus mainly on the antiplatelet effect and the inherent hemorrhagic risk that some of these drugs can pose to users.

Since NSAID is a heterogeneous class of drugs, it is important to differentiate the weight that each drug has on the bleeding risk. ASA, for instance, carries a higher risk of bleeding; celecoxib, on the other hand, has been associated with a lower bleeding risk, probably due to its different pharmacodynamic properties [68].

In sports medicine, other drugs such as diclofenac and ibuprofen are often used for their anti-inflammatory and pain relief. A recent RCT failed to show an interference of diclofenac on platelet function and the bleeding risk in patients undergoing CABG, taking clinically administered doses [69]. Nonetheless, other studies underscore the increased bleeding risk that these drugs pose to the patients, especially when used concomitantly with other antithrombotic drugs [70,71], paying particular caution when discussing eligibility to sports practice, RTP, and sports recommendations. In the case of diclofenac and ibuprofen, they have a clinical indication when a potential increase in bleeding risk exists, oral and parenteral administration may be avoided, and topical application minimizing systemic absorption should be preferred [72]. Furthermore, it is suggested to consider regular paracetamol as the first-line treatment for acute and chronic musculoskeletal pain due to comparable analgesic efficacy with NSAID but a lower side effect profile [73].

Gastrointestinal bleeding is a common adverse effect of NSAIDs due to the inhibition of cyclooxygenase-1 and the subsequent reduction in prostaglandin in the mucosal lining of the stomach. In athletes, gastrointestinal bleeding can be caused not only by NSAID use but also by different pathophysiological mechanisms, especially in long-distance runners, such as splanchnic hypoperfusion and mechanical traumas [74]. However, this is a growing field of research, and specific indications are not fully available yet, thus requiring further studies.

Therefore, the potential NSAID-induced increased risk of bleeding should be considered when prescribing SAPT or DAPT in competitive athletes, and they should be educated and informed in detail about the potential negative effects of this pharmacological class when they are taking antiplatelet therapy (see Table 1).

Another factor that must be taken into account when managing antiplatelet therapy in athletes is the potential abuse of anabolic androgenic steroids (AAS). Their use has been linked to an increased risk of acute coronary events, which may further complicate the management of antithrombotic therapy [75,76]. AAS can accelerate atherosclerosis, promote thrombosis, and contribute to endothelial dysfunction, thereby increasing the likelihood of myocardial infarction, even in young athletes [77]. However, it is important to underline that most of the current knowledge on this topic comes from case reports or small studies, highlighting the need for further research to establish clearer clinical guidelines.

## 2. Conclusions

Athletes with an indication for anticoagulation or antiplatelet therapy should be carefully evaluated, balancing the individual ischemic and bleeding risk profile and the intrinsic risk of sports activities such as contact sports. Although athletes should receive the conventional antithrombotic regimen recommended for the general population, the default approach for antithrombotic therapy could be personalized in selected cases with low ischemic risk. For instance, a shorter duration of DAPT may benefit athletes by lowering the bleeding risk associated with antiplatelet therapy, potentially enabling a safer and earlier return to play. However, sports eligibility is influenced not only by therapy but mainly by the clinical presentation and characteristics of the cardiovascular disorder experienced by the athlete. Additionally, since athletes abuse NSAIDs, given the intrinsic antiplatelet activity of most of these drugs, careful recommendations must be added. Further studies are needed to validate the safety and efficacy of specific approaches, such as intermitting dosing strategies, in the specific population of competitive athletes.

## Figures and Tables

**Table 1:** Take-home messages on the management of antiplatelet and anticoagulation therapy in competitive athletes.

**General considerations**
The estimation of the bleeding risk in competitive athletes is based on two components: (a) the individual risk; (b) the sport-specific risk. The latter is related to the type of sport practiced (e.g., traumatic or contact sports) and is determined by several factors (e.g., the type and frequency of collisions, the utilization of different protections and the hardness of the soil or the ball).
The default approach of antithrombotic treatment can be personalized in selected cases. However, personalization should be balanced with offering the best therapy possible, irrespective of athletic status.
The use and abuse of NSAIDs should also be considered when prescribing DAPT and SAPT in professional and amateur competitive athletes to assess a real bleeding risk and to obtain a correct risk stratification.
The athlete should be informed and educated about the potential negative effects of NSAIDs.
In case NSAIDs are indicated and the athlete is under SAPT, DAPT, or is anticoagulated, the treatment duration should be as short as possible, and topical administration should be preferred for drugs associated with increased bleeding risk in order to minimize the systemic absorption.
**Acute and chronic coronary syndromes**
The procedure of revascularization and DAPT duration should be adapted in competitive athletes, when possible, to allow a safe return to sports practice.
During DAPT, sports at risk for trauma must be avoided because of the hemorrhagic risk, and all guidelines and protocols suggest suspending activities with expected impacts, considering the high hemorrhagic risk.
In selected cases at low ischemic risk, DAPT should be shortened to allow a safe return to sports practice, always balancing the benefits associated with standard treatment
After an ACS, a large proportion of the athletes are no longer eligible to practice competitive sports, irrespective of antiplatelet and anticoagulation therapy.
It is highly recommended to prescribe a structured and tailored exercise program aiming to improve the cardiovascular risk profile and cardiovascular outcomes in patients with acute and chronic coronary syndromes.
**Atrial septal defect and patent foramen ovale closure**
DAPT is indicated from 1 to 6 months after the procedure.
It can be shortened to 1 month in selected cases after a successful procedure
After a successful procedure, athletes are eligible for sports competition with an interval between 3 and 6 months.
After PFO closure, all competitive sports, including scuba diving, are allowed in the absence of residual shunts. Conversely, scuba diving is not allowed in case of residual shunts due to a risk of paradoxical embolism.
After PFO closure, SAPT should be stopped after 3 months.
After ASD closure, SAPT should be stopped after 6 months in selected cases with relevant bleeding risk, also considering the type of sports practiced (e.g., contact or traumatic sports), to allow a safe sports practice.
The device dislocation is a rare but relevant complication. A strict follow-up is mandatory in athletes engaged in contact or traumatic sports.
**Atrial fibrillation and deep vein thrombosis**
Antithrombotic therapy is indicated based on the criteria and scores recommended for the general population, even if, typically, this treatment is not indicated in competitive athletes because of the low-very low ischemic risk.
Sports with direct bodily contact or prone to trauma are not recommended for exercising individuals with AF who are anticoagulated.
Athletes with paroxysmal AF and not anticoagulated, in the absence of structural and electrical heart disease, are eligible for all competitive sports, except for symptomatic individuals with pre-syncope or syncope.
AF ablation should be indicated in competitive athletes with a high rate of success.
Intermitting dosing strategies have been proposed to accelerate the return-to-play of some athletes 3 months after DVT. Further data are needed to confirm the efficacy of this strategy.

DAPT, double antiplatelet therapy; SAPT, single antiplatelet therapy; NSAIDs, nonsteroidal anti-inflammatory drugs; ACS, acute coronary syndrome; PFO, patent foramen ovale; ASD, atrial septal defect; AF, atrial fibrillation; DVT, deep venous thrombosis.

## Data Availability

No new data were created or analyzed in this study. Data sharing is not applicable to this article.
